# COVID Obesity: A One-Year Narrative Review

**DOI:** 10.3390/nu13062060

**Published:** 2021-06-16

**Authors:** Diana L. Palacios Ovalle, Susana Rodrigo-Cano, Aránzazu González, Carla Soler, Ana I. Catalá-Gregori, J. Francisco Merino-Torres, Jose M. Soriano

**Affiliations:** 1 Food & Health Lab, Institute of Materials Science, University of Valencia, Paterna, 46980 Valencia, Spain; diapao@alumni.uv.es (D.L.P.O.); rodcasu@hotmail.com (S.R.-C.); aranzazugonzalez999@gmail.com (A.G.); carla.soler@uv.es (C.S.); 2 Joint Research Unit on Endocrinology, Nutrition and Clinical Dietetics, University of Valencia-Health Research Institute La Fe, 46026 Valencia, Spain; catala_anagre@gva.es (A.I.C.-G.); merino_jfr@gva.es (J.F.M.-T.); 3 Department of Endocrinology and Nutrition, University and Polytechnic Hospital La Fe, 46026 Valencia, Spain

**Keywords:** COVID-19, BMI, obesity, overweight, nutrition, COVID obesity

## Abstract

On 11 March 2020, coronavirus disease 2019 (COVID-19) was declared a pandemic by the World Health Organization (WHO). This study focuses on a narrative review about the illness during the first year of the pandemic in relation to obesity. Databases were used to search studies published up to 8 December 2020. In total, 4430 articles and other scientific literature were found, and 24 articles were included in this one-year narrative review. The mean BMI value of severe COVID-19 patients ranged from 24.5 to 33.4 kg/m^2^, versus <18.5 to 24.3 kg/m^2^ for non-severe patients. Articles using the terms obesity or overweight without indicating the BMI value in these patients were common, but this is not useful, as the anthropometric parameters, when not defined by this index, are confusing due to the classification being different in the West compared to among Asian and Korean criteria-based adults. We proposed a new term, called COVID obesity, to define the importance of this anthropometric parameter, among others, in relation with this pandemic.

## 1. Introduction

A new epidemic started on 8 December 2019 in Wuhan (Hubei Province, China), where several cases of severe pneumonia of undetermined etiology were reported based on fever (>38 °C), cough, fatigue, muscle pain, leukopenia, lymphopenia, and radiographic imaging consistent with pneumonia, which could develop into acute respiratory distress syndrome, metabolic acidosis, septic shock, coagulation dysfunction, organ failure (such as liver, kidney, and heart failure), and death [1]. This disease is caused by a new strain of coronavirus, pre-defined as novel coronavirus (2019-nCoV), which causes upper respiratory tract infections in humans [2], and which, along with severe acute respiratory syndrome coronavirus (SARS-CoV) [3] and Middle East respiratory syndrome coronavirus (MERS-CoV) [4], evolved in the twenty-first century [5]. On 11 February 2020, the new coronavirus was renamed SARS-CoV-2 from 2019-nCoV (Coronaviridae Study Group of the International Committee on Taxonomy of Viruses, 2020), and the disease caused by SARS-CoV-2 was called coronavirus disease 2019 (COVID-19) [6]. The World Health Organization (WHO) declared COVID-19 a pandemic on 11 March 2020 [7], pointing to the over 118,000 cases of the illness, with a global death toll that had reached 4291 people in over 114 countries and territories around the world and a sustained risk of further global spread [8]. Currently, the WHO confirmed on 4 June 2021 that there were 171,782,908 confirmed cases of COVID-19, including 3,698,621 deaths [9]. However, history allows us to learn lessons from the past, especially with diseases of other viruses causing pneumonia in relation to body mass index (BMI: weight/height^2^ (kg/m^2^)), which has been used as a disease-associated factor. BMI is an anthropometric parameter used to assess malnutrition risk, but has been observed as a risk factor in MERS-CoV [10], H1N1 influenza [11], and avian influenza H7N9 virus [12]. Currently, severe COVID-19 patients seem to have a higher BMI or obesity [13,14,15,16], therefore, this index should be considered a factor that could aggravate the disease. The aim of this narrative review was to analyze the anthropometric parameters in patients with COVID-19 one year after the origin of this disease.

## 2. Materials and Methods

This narrative review searched for high nutritional status (obesity and overweightness) and its parameter (BMI) conducted in patients with COVID-19 using the Preferred Reporting Items for Systematic Reviews and Meta-Analyses (PRISMA) statement [17]. The search was conducted using the WHO COVID-19 database of publications, PubMed, Scopus, Embase, and Google Scholar. We included English and other languages that were translated using the freely available web-based Google Translator (Google, Inc., Mountain View, CA); in cases of doubt for the manuscripts written in Asian languages, the help of the Confucius Institute from the University of Valencia was used before the full papers were reviewed. Key search terms were determined in accordance with the Population, Intervention, Comparator, Outcome (PICO) framework [18], and this was conducted to find studies by including two main searches based on the coronavirus as a causal agent or disease and an anthropometric parameter: “novel coronavirus” or “2019 Novel Coronavirus” or “2019-nCoV” or “SARS-CoV-2” or “COVID-19” or “coronavirus disease 2019” AND “body mass index” or “BMI” or “weight” or “height” or “obesity” or “obese” or “overweight” or “underweight” or “normal weight” or “malnutrition” or “nutritional status”. The search was carried out from home confinement on 8 December 2020. Classifying BMI/obesity in adults according to international values was observed by the WHO [19,20,21] to perhaps not be appropriate for Asian and Korean populations. In fact, Asian populations have a higher body fat deposit at a lower BMI than Caucasians, but show an increasing trend toward obesity. For this reason, there are different cutoffs for international, Asian, and Korean populations. The classification of anthropometric parameters was carried out in accordance with the guidelines from the WHO [19,20] and Seo et al. [21] for international, Asian, and Korean criteria-based adults, respectively (Table 1).

As inclusion criteria, we required that the study samples were humans, and that the articles were full text and available in any language. On the other hand, as exclusion criteria, we excluded studies in which an anthropometric parameter was not used, as well as conference abstracts, simulation studies, unpublished data, and articles without full texts. Three teams of paired reviewers (D.L.P.O., S.R.C., J.M.S., A.G., A.I.C.-G, and J.F.T-M) with expertise in medical and health evaluations and training in research methodology independently screened the titles, abstracts, and full texts for eligibility, assessed generalizability, and collected data from each eligible study using a standardized pilot-tested form with detailed instructions, which included the following information: author name, type of study, number of patients, age, sex, country, anthropometric parameter, and main outcomes. Any disagreements were resolved by a third researcher (C.S.). Based on information from the National Heart, Lung and Blood Institute [22], the validity of each included study was assessed using nine items, to which the answer was affirmative (+), negative (–), or other, including “cannot determine”, “not applicable”, and “not reported”, which were considered unclear (?) answers. We have classified the studies using a rating of good (7–9), fair (4–6), or poor (≤3) for each individual study. The study quality was determined by comparing rating agreement, with consensus required among the reviewers. Discrepancies in study quality rating were reconciled via discussion of the individual items on the ratings checklist to arrive to an agreement on the quality indicator.

## 3. Results

**Figure 1:**
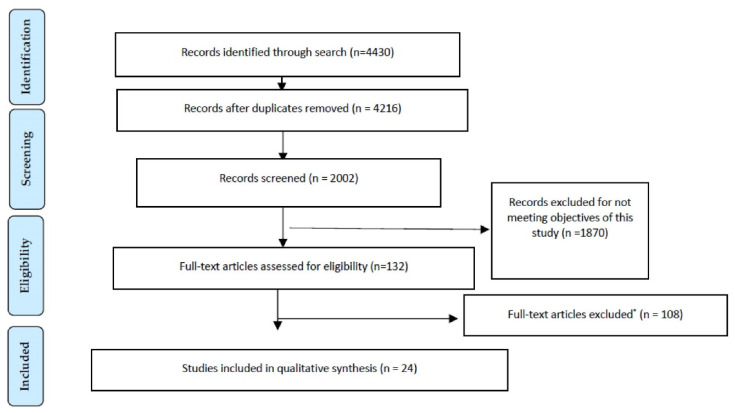
PRISMA flow diagram for studies retrieved through the searching and selection process. * Reasons for exclusion of articles are presented in the Materials and Methods section.

Out of 4430 papers identified in the initial search, 24 studies were included; 2214 records were not screened due to being unrelated articles by analyzing their titles and abstracts. The PRISMA flow diagram for narrative reviews is presented in Figure 1. The quality rating of the reviewed literature, found by applying the National Heart, Lung and Blood Institute criteria, was fair (Table 2), which is similar to the study of Salehi et al. [23], who carried out a systematic review of imaging findings in 919 COVID-19 patients and observed that the methodologic quality of the studies was generally fair. In total, we integrated 24 studies relevant to the aim of this review. All articles were on studies carried out in China (*n* = 11), Korea (*n* = 2), France (*n* = 1), the USA (*n* = 4), Italy (*n* = 1), Spain (*n* = 4), and Mexico (*n* = 1). Table 3 shows the summary of these studies according to the term BMI extracted from the reviewed articles.

### 3.1. China

Li et al. [26] reflected on a case report of a Chinese man (59 years old) with no history of Southern China seafood market contact who had experienced hypertension for the past 20 years (treated using oral valsartan and bisoprolol fumarate tablets as medication), coronary heart disease for the last six years (treated using oral bisoprolol fumarate tablets and betaloc), and endured a long-term anti-rejection treatment (using cyclosporine and bradinine) for five years after right kidney transplantation. The evolution of the disease was as follows: fever (38.2 °C), dyspnea, headache, myodynia, a ground glass shadow observed in a CT scan of the lung in the upper and lower regions of the left lung, cough and expectoration (with a small amount of white sputum), nausea without vomiting, abdominal pain, diarrhea, exertional dyspnea without obvious aggravation, hemoptysis, chest pain, acid reflux, heartburn, chest distress and suffocation, progressive dyspnea, progressive deterioration of renal function while undergoing treatment, delirium (on the fifth day after admission), loss of consciousness, lowered blood pressure and heart rhythm, and death. The patient had a BMI of 26.6 kg/m^2^, putting him in obesity class I [20].

On the other hand, Liu et al. [27] studied medical workers (aged 21–59 years) infected by COVID-19 from the affiliated hospital of Jianghan University, Wuhan, China, between 3 January 2020 and 11 January 2020, and observed a significant difference in BMI. It was 22.0 ± 1.3 (normal weight) versus 27.0 ± 2.5 (obesity class I) kg/m^2^ among the 26 common type and four severe case patients, respectively. Liu et al. [28] indicated that the infected medical staff from Union Hospital, Wuhan, between 16 January 2020 to 15 February 2020, had a BMI of < 24 (*n* = 33/37) and ≥ 24 (*n* = 4/37) kg/m^2^ among the group of patients with symptom onset for 10 days or less, and < 24 (*n* = 22/25) and ≥ 24 (*n* = 3/25) kg/m^2^ for those with symptoms for more than 10 days, respectively, by the time of admission. However, these authors indicated that the value of ≥ 24 kg/m^2^ was overweight, and this was a mistake due to the fact that the values for overweightness for Asian adults, established by the WHO [20], range from 23.0 to 24.9 kg/m^2^.

Yang et al. [29] carried out at retrospective cohort study of 149 positive patients (45.6% and 54.4% female and male, respectively, aged 45.11 ± 13.35, from 17 January 2020 to 10 February 2020 in three tertiary hospitals of Wenzhou, Zhejiang province (China)) and found that the value of the BMI was 23.75 ± 4.54 kg/m^2^ (overweight). Peng et al. [30] analyzed 112 patients with cardiovascular disease in the western district of Union Hospital in Wuhan, from 20 January 2020 to 15 February 2020, and found that the average (first and third quartile) BMI of the critical group (*n* = 16) was significantly higher than that of the general group (*n* = 96) at 25.5 (23.0 and 27.5) versus 22.0 (20.0 and 24.0) kg/m^2^. These authors observed that patients with BMI > 25 kg/m^2^ (obesity class I) were significantly more likely to be non-survivors (88.24%, *n* = 15/17) versus survivors (18.95%, *n* = 18/95). 

Cai et al. [31] reviewed a study in the Third People’s Hospital of Shenzhen, Guangdong, China, from 11 January 2020 to 6 February 2020 with 298 patients (aged 33–61 years) with COVID-19, and no significant differences were found between non-severe (*n* = 240) and severe (*n* = 58) patients, with the value of the BMI being 22.9 (normal weight) and 24.5 kg/m^2^ (overweight), respectively. Furthermore, these authors demonstrated that gender, BMI, and the antiviral agents lopinavir/ritonavir or favipiravir were not independent prognostic factors for virus clearance.

Interestingly, Huang et al. [32] carried out a multicenter retrospective cohort study in 12 hospitals from Jiangsu, China, between 24 January and 23 February 2020, detecting severe patients (*n* = 60) out of 631 infected cases with COVID-19, with the average BMI among them being 25.0 ± 3.3 kg/m², but no significant differences were found between improved (*n* = 52) and impaired (*n* = 8) patients in terms of BMI (25.2 ± 3.4 and 23.7 ± 2.5 kg/m^2^, respectively). The same author published another article [48] in which a retrospective cohort study was carried out between 11 December 2019 and 29 January 2020, but they did not include or did not have the value of the BMI.

On the other hand, a multi-centered, retrospective, observational study was carried out by Xu et al. [33], where 45 critically ill patients with COVID-19 (35.6% and 64.4% female and male, respectively, aged 56.7 ± 15.4) identified in seven intensive care units in Guangdong, China. The average values for BMI were 23.2 (overweight) and 25.0 kg/m² (obesity class I) for patients who underwent intubation and non-intubation, respectively.

### 3.2. Korea

Lim et al. [24] reported for the first time a case of transmission of COVID-19 outside China: a Korean man who was living in Wuhan (China) and entered Korea on 20 January 2020. He is considered the index patient who transmitted the coronavirus at a restaurant to another person (confirmed on 30 January 2020). This patient transmitted the virus to his family (spouse and son) and a friend. The index patient was 54 years old with a BMI of 25.7 kg/m^2^, in obesity class I [24], and lopinavir/ritonavir was used as the treatment. A reduction of viral loads and improvement of clinical symptoms during the treatment was observed. Kim et al. [25] described the first patient with 2019-nCoV pneumonia in Korea, a 35-year-old woman admitted to the hospital on 19 January 2020, who was obese with a BMI of 33.4 kg/m^2^ (obesity class II [21]), but otherwise healthy.

### 3.3. France

Simonnet et al. [35] carried out a retrospective cohort study analyzing BMI and the requirement for invasive mechanical ventilation in 124 COVID-19 patients admitted into intensive care at Roger Salengro Hospital, at the “Centre Hospitalier Universitaire de Lille” (Lille, France). These authors reflected that the disease severity was associated with increased BMI, and was maximal in patients with a BMI ≥ 35 kg/m^2^. A higher likelihood of needing invasive mechanical ventilation was noted for this BMI, independent of age, sex, diabetes, and hypertension.

### 3.4. USA

New York was one of the sites most affected by the pandemic, where a retrospective cohort study was carried out by Palaiodimos et al. [36], including the first 200 patients in a tertiary medical center called Montefiore. This study aimed to evaluate the characteristics of the hospitalized patients, finding that severe obesity (≥ 35 kg / m^2^), older age, and male sex were independently associated with mortality, the need for intubation, and oxygen requirement during hospital stay. Nilles et al. [38] conducted a prospective cohort study with 4469 non-hospitalized employees of Space Exploration Technologies Corporation; the most important finding was the association of obesity with the increase in symptoms of mild COVID-19 infections, otherwise not having an association with the increase of the susceptibility to infection by COVID-19. There were also no different immunological characteristics between obese and non-obese patients. Some studies suggest that obesity may be a risk factor in younger people. One of them, with 3615 patients, found that obesity was significantly related to hospital admission and intensive care unit (ICU) admission among patients under 60 years of age [37]. In the same case, a retrospective cohort study strongly associated obesity with intubation or mortality among adults younger than 65 years, but not among those older than 65 years [39].

### 3.5. Spain

Fernandez García et al. [42] reflected in a study with 49 patients from the internal medicine hospital ward for COVID-19 infection at the Toledo hospital that diabetes, but not obesity, was defined as a factor that influenced admission to the ICU. Casas-Rojo et al. [44] carried out an observational study based on the SEMI-COVID registry, a retrospective cohort that includes patients discharged or deceased after confirmed COVID-19 in 150 hospitals in Spain, including a sample from March 27 to June 30, where a high percentage of patients with comorbidities were shown, the main ones being arterial hypertension, dyslipidemia, obesity, and diabetes mellitus. Ferrando et al. [43] described the clinical characteristics of patients admitted to the ICU; obesity was mentioned among the most prevalent comorbidities. Rosales Castillo et al. [45] showed a persistence of signs and symptoms after being discharged for COVID-19, and, within their population, 37.3% were overweight and 41% were obese, a considerable percentage, also presenting in their results an average BMI within overweight ranges.

### 3.6. Italy

Halasz et al. [46] carried out a retrospective study in which a cohort of patients was analyzed after their admission to the ICU at the Saliceto hospital in Piancenza, Italy, finding a link between overweightness (25–30 kg/m^2^) and obesity (>30 kg/m^2^) and the risk of developing severe acute respiratory distress syndrome (ARDS). Their data showed that not only obese patients, but also overweight patients had a higher risk of entering the ICU, and that severe obesity was associated with increased mortality and invasive ventilation.

### 3.7. Mexico

Herrera García et al. [47] observed that a population in a pneumology outpatient clinic had a value of 28 ± 3 kg/m^2^ who survived, but they were affected with “long COVID”, which is a term used to describe the illness in people who have either recovered from COVID-19, but are still reporting lasting effects of the infection, or have had the usual symptoms for far longer than would be expected.

## 4. Discussion

To date, only 24 articles related to BMI and COVID-19 have been published. We observed that BMI values among severe COVID-19 patients ranged from 24.5 to 33.4 kg/m^2^ versus those among non-severe patients, which ranged from < 18.5 to 24.3 kg/m^2^. Liu et al. [28] indicated that a BMI ≥ 24 kg/m^2^ on admission was an unfavorable factor for discharge, but if a statistical difference of *p* < 0.005 among the BMI values is required for it to be considered a potential risk factor for predicting progression to severe disease, only nine [27,30,31,36,37,39,41,42,46] of the studies met this criteria. Accordingly, we proposed that a BMI ≥ 24.9 kg/m^2^, which is indicative of normal weight [19] or being overweight [20,21], should be the cutoff for patients to move from non-severe to severe status. Furthermore, our group thinks that the use of another related term in the bibliography, such as obesity, is unclear in relation to this disease, as the authors indicated that some patients had this anthropometric parameter, but it was not reflected in the BMI value, according to the BMI cutoffs. This is mainly because the BMI classification for Asians [20], established from restricted numbers of prevalence studies, but not from mortality data, is different from the recommended WHO BMI cutoffs for the West [19], and even from cutoffs for Koreans [21]. Surprisingly, some studies that our group excluded from this review mentioned obesity without a detailed specification of the BMI value. We analyzed how these articles were reflected in Table 4.

Liao et al. [51] observed reports of overweightness/obesity, without giving BMI values, among 28.6% and 40.6% of adolescents (10–24 years of age) and young adults (25–35 years of age), respectively, who were infected with COVID-19 and hospitalized in China. In a single center, Deng et al. [50] did not indicate the values of BMI, but indicated no significant differences in the presence of obesity. It is unknown whether they were referring to the values for the West [19] or Asian criteria-based [20] adults. In the USA, 33.3% of residents and 3% of visitors infected by coronavirus in a long-term residential care facility were obese, but no infected healthcare personnel had this anthropometric parameter [49]. Among the first European cases, one was reported to be obese, but they did not provide BMI values for the rest of the cases [53]. In the USA, an obese woman with other pathologies, including coronary artery disease, insulin-dependent type II diabetes mellitus, chronic kidney disease, hypertension, and congestive heart failure, was infected with COVID-19 [49]. The term obesity is used in the primary case pre-existing condition application form entitled Surface Sampling of COVID-19: A Practical “How To” Protocol for Health Care and Public Health Professionals [54]. Zhong et al. [55] described in a quick online cross-sectional questionnaire of knowledge, attitudes, and practice towards COVID-19, in the item K4, that “Not all persons with COVID-2019 will develop to severe cases. Only those who are elderly, have chronic illnesses, and are obese are more likely to be severe cases.” Considering that the measurement of BMI is the usual parameter in the triage system from hospital emergency departments [56,57], in our view, this index should be added to all studies of COVID-19 for two reasons: the possible risk factor of BMI in relation with other virus diseases [10,11,12], and the association of pneumonia (which is a symptom of COVID-19) with BMI [12,13,14,15]. We must keep in mind that COVID-19 is spread from person to person by the same mechanism as other common cold or influenza viruses [58]. Several authors [59,60] found that obesity was a risk factor in influenza-like illness. Moser et al. [61] suggested that being underweight or morbidly obese in adults, even with no associated chronic conditions, can increase the risk of influenza-related complications, and could influence the mortality and transmission of the influenza virus [62,63]. Dietz and Santos-Burgoa [64] emphasized the need for increased vigilance, a priority around detection and testing, and aggressive therapy for patients with obesity and COVID-19 infections. Nevertheless, the use of BMI in COVID-19 can be confusing. Jin et al. [65] developed a core outcome set for clinical trials on COVID-19 and included the BMI in the first round of the Delphi survey, but deleted it in the second round. Arnold et al. [66] established a BMI value of 25 and 28 kg/m² for patients with disease caused by endemic human coronavirus and influenza virus, respectively, and observed statistically significant impacts on comorbidities based on the BMI, but not obesity (BMI > 30 kg/m²).

The evolution of this pandemic has been reported to begin with asymptomatic infection to mild illness, and severe or fatal illness being the last stage, with the need for intensive care and respiratory support, in which patients receive high-flow oxygen therapy, mechanical ventilation, advanced organ support with endotracheal intubation, and mechanical ventilation or extracorporeal membrane oxygenation [67,68,69]. If focusing on patients with COVID-19 who were admitted to the ICU, there is only one study [33] where the BMI values were compared, without statistical analysis, between intubated (*n* = 20) and non-intubated (*n* = 25) patients. Cai et al. [31] illustrated that the BMI seems to have little effect on the progress of the disease, and Ji et al. [34] revealed statistically non-significant differences between stable non-severe and progressively severe patients. However, several authors found different results; Liu et al. [27] and Peng et al. [30] observed that the BMI of severely ill patients was significantly higher than the values of common patients. For viral load, Liu et al. [70] demonstrated that this analytical parameter is higher in patients with more severe disease, based on the amount of virus exposure and known infectious dose, which increased the severity of the illness. Cai et al. [31] documented that BMI was not an independent prognostic factor for virus clearance. On the other hand, the term of hospital discharge or release from home isolation was defined [71] when three of the following applied: (i) afebrile for > 3 days and improved respiratory symptoms; (ii) resolution of lung involvement demonstrated by computed tomography of the chest; and (iii) two consecutive (with sampling intervals ≥ 24 h) negative RT-PCR tests for respiratory tract samples. The study by Liu et al. [28] demonstrated that a higher BMI (≥ 24 kg/m^2^) was an unfavorable factor for discharge among infected medical staff.

The importance of considering BMI in relation to COVID-19 has larger implications than explained above: underlying comorbidities can aggravate the disease. China [72,73] and the European Union [74] have suggested that hypertension, respiratory system disease, and cardiovascular diseases are problematic; Italy [75] reflected that hypertension, diabetes, ischemic heart disease, atrial fibrillation, and cancer diagnosed in the last five years complicate the disease. Ghana [76] and other tropical and sub-tropical areas have detected cardiovascular disease, diabetes, chronic respiratory disease, hypertension, cancer, and *Plasmodium falciparum* malaria as underlying conditions of concern, and Latin American countries [77] have indicated obesity, diabetes mellitus, and hypertension as important. Shi et al. [78] observed that the mortality of diabetic patients with COVID-19 was 35.4%, due to a higher likelihood of suffering from multi-organ dysfunction and secondary infection. The use of our proposal of BMI ≥ 24.9 kg/m^2^ as a risk factor in COVID-19 could be applied to the three groups defined by Wang et al. [79], with a high risk factor indicated when the BMI ≥ 34.9 kg/m^2^. In fact, a high BMI can worsen COVID-19 symptoms due to several circumstances, such as an increase in inflammatory cytokines and decrease in expiratory reserve volume, functional capacity, and respiratory system compliance [80].

On the other hand, the use of nutritional support therapy during hospitalization is key in strengthening the patients. Li et al. [81] found that BMI had no effect on in-hospital mortality, but may be closely correlated with prolonged intubation for patients. Xu et al. [82] indicated that nutritional and gastrointestinal function should be assessed for all patients. Li et al. [83] observed a decline of albumin, presumably to the exuberant protein synthesis caused by infected patients’ hypermetabolic state, associated with fever, malnutrition, and low caloric intake, which can reduce BMI values through weight loss. A case reflected a weight loss of around 8 kg at the time of the patient’s discharge from the hospital [84]. Let us not forget that prolonged ICU stays for COVID-19 can indirectly worsen or cause malnutrition. It is worth noting that the European Society for Clinical Nutrition and Metabolism (ESPEN) has been carried out a concise guidance for nutritional management of infected patients in the ICU setting or who are of older age and polymorbidity status [85], and the French-speaking Society for Clinical Nutrition and Metabolism (SFNCM)’s Home Artificial Nutrition Committee has elaborated some recommendations for home artificial nutrition for chronic and fragile COVID-19 patients [86]. Furthermore, several authors [87,88] have developed provisional guidance on nutrition and diet for managing pediatric inflammatory bowel and kidney diseases, respectively.

The limitations of this study include the small number of available studies for review and different sample sizes (from one to 15.111 patients). A meta-analysis was not conducted due to the varied interventions in the reported studies.

## 5. Conclusions

In conclusion, the use of the BMI is a key tool to define the anthropometric parameters in infected COVID-19 patients, and should be added in the triage system for hospital emergency departments. This review explores the few published articles that consider this index, as well as the confusing term obesity if the BMI was not incorporated into the manuscripts. Clinicians should keep a patient’s BMI in mind when evaluating risk and deciding on a course of treatment for COVID-19 to improve both short- and long-term prognoses. According to these results, we propose a new term, COVID obesity, which is defined as the relationship between obesity and COVID-19, with implications in terms of the gravity of the illness worldwide. 

## Figures and Tables

**Table 1:** Classification of body mass index (BMI; kg/m^2^) and the risk of co-morbidities in international, Asian, and Korean criteria-based adults according to the WHO [19,20] and Seo et al. [21], respectively.

Anthropometric Parameters	International Adults		Asian Criteria-Based Adults		Korean Criteria-Based Adults	
	BMI (kg/m^2^)	Risk of Co-Morbidities	BMI (kg/m^2^)	Risk of Co-Morbidities	BMI (kg/m^2^)	Risk of Co-Morbidities
Underweight	<18.5	Lower (other health risk)	<18.5	Lower (other health risk)	<18.5	Low ^a^/average ^b^
Normal weight	18.5–24.9	Average	18.5–22.9	Average	18.5–22.9	Average ^a^/increased ^b^
Overweight (pre-obesity)	25.0–29.9	Increased	23.0–24.9	Increased	23.0–24.9	Increased ^a^/moderate ^b^
Obesity class I	30.0–34.9	Moderate	25.0–29.9	Moderate	25.0–29.9	High ^a^/severe ^b^
Obesity class II	35.0–39.9	Severe	≥30.0	Severe	30.0–34.9	Moderate ^a^/very severe ^b^
Obesity class III	≥40.0	Very severe	n.a.	n.a.	≥35	Severe^a^/very severe ^b^

^a^ Risk of comorbidity according to abdominal obesity < 90 cm (men) and < 85 cm (women). ^b^ Risk of comorbidity according to abdominal obesity ≥ 90 cm (men) and ≥ 85 cm (women); n.a., not applicable.

**Table 2:** Methodological quality assessment for studies published on BMI and COVID-19 according to the criteria of the National Heart, Lung and Blood Institute [22].

Reference/Item ^a^	1	2	3	4	5	6	7	8	9	Quality Rating ^b^
[24]	+	+	?	?	+	+	+	?	+	6
[25]	+	+	?	?	+	+	+	?	+	6
[26]	+	+	?	?	+	+	+	?	+	6
[27]	+	+	?	?	+	+	+	+	+	6
[28]	+	+	?	?	+	+	+	+	+	6
[29]	+	+	?	?	?	+	?	+	+	5
[30]	+	+	?	?	?	+	?	?	+	4
[31]	+	+	?	?	?	+	?	?	+	4
[32]	+	+	?	?	?	+	?	?	+	5
[33]	+	+	?	?	?	+	?	-	+	4
[34]	+	+	?	?	?	+	?	?	+	4
[35]	+	+	?	?	+	+	+	+	+	7
[36]	+	+	?	?	?	+	?	+	+	5
[37]	+	+	?	?	?	+	+	+	+	6
[38]	+	+	?	?	?	+	+	+	+	6
[39]	+	+	?	?	+	+	+	+	+	7
[40]	+	+	?	?	+	+	+	+	+	7
[41]	+	+	?	?	+	+	?	+	+	6
[42]	+	+	?	?	?	+	?	?	+	4
[43]	+	+	?	?	+	+	+	+	+	7
[44]	+	+	?	?	+	+	+	?	+	6
[45]	+	+	?	?	?	+	?	?	+	4
[46]	+	+	?	?	?	+	+	?	+	5
[47]	+	+	?	?	+	+	+	?	+	6

Affirmative (+), negative (–), or other, including “cannot determine”, “not applicable”, and “not reported”, which were considered unclear (?) answers. ^a^ Items from the National Heart, Lung and Blood Institute [22] were: 1 = was the study question or objective clearly stated?; 2 = was the study population clearly and fully described, including a case definition?; 3 = were the cases consecutive?; 4 = were the subjects comparable?; 5 = was the intervention clearly described?; 6 = were the outcome measures clearly defined, valid, reliable, and implemented consistently across all study participants?; 7 = was the length of follow-up adequate?; 8 = were the statistical methods well-described?; and 9 = were the results well-described? ^b^ Quality rating [21] was good (7–9), fair (4–6), or poor (≤3).

**Table 3:** Summary of articles with data on BMI in COVID-19 infected patients.

No. of Patients	Age	Sex	Country	BMI (kg/m^2^)/Anthropometric Parameters [18,19,20]	References
1	54	Male (M)	Korea	25.7/Obesity class I	[24]
1	35	Female (F)	Korea	33.4/Obesity class II	[25]
1	59	M	China	26.6/Obesity class I	[26]
30	21–59	F (66.7%)/M (33.3%)	China	22.0 ± 1.3/Normal weight ^a^ 27.0 ± 2.5/Obesity class I ^b^	[27]
64	35.0 (average)	F (64.0%)/M (36.0%)	China	<24 (89.2%) and ≥ 24 (10.8%) ^c^ <24 (88.0%) and ≥ 24 (12.0%) ^d^	[28]
149	45.11 ± 13.35	F (45.6%)/M (54.4%)	China	23.7 ± 4.5/Overweight	[29]
112	62.0 (average)	F (52.7%)/M (47.3%)	China	22.0 (22.0–24.0)/Normal weight ^e^ 25.5 (23.0–27.5)/Obesity class I ^f^	[30]
298	33–61	F (50.0%)/M (50.0%)	China	22.9 (20.6–25.2)/Normal weight ^g^ 24.5 (22.0–27.8)/Overweight ^h^	[31]
60	57 (average)	F (41.7%)/M (58.3%)	China	25.0 ± 3.3/Obesity class I	[32]
45	56.7 ± 15.4	F (35.6%)/M (64.4%)	China	23.2 (21.4–25.3)/Overweight ^i^ 25.2 (22.9–26.9)/Obesity class I ^j^	[33]
49	43.6 ± 17.1	F (36.7%)/M (63.3%)	China	24.3 ± 3.6/Overweight ^k^ 26.4 ± 2.8/Obesity class I ^l^	[34]
124	51–70	F (27.0%)/M (73.0%)	France	31.1 (27.3–37.5)/Obesity class I ^m^ 27.0 (25.3–30.8)/Overweight I ^n^	[35]
200	64 (50–73.5)	F (51.0%)/M (49.0%)	USA	30.0 (26–35) Obesity class I	[36]
3615	≥0 to <60 (mention two groups)	Both	USA	30–34 (21%) and ≥ 35 (16%)	[37]
4469	>18	F (600)/M (3730) Not reported (139)	USA	27.1 ± 5.4 Overweight	[38]
2466	67 (54–78)	F (42%)/M (58%)	USA	27.9 (24.3–32.6)/Overweight	[39]
383	28–62	F(52.2%)/M (47.8%)	China	<18.5 (4.2%) 18.5–23.9 (53.1%) 24.0–27.9 (32.0%) >28.0 (10.7%)	[40]
96	17–62	F (45.0%)/M (54.9%)	China	<24 (61.45%) and ≥24 (38.55%)	[41]
49	60.3 ± 11.8	F (42.8%)/M (57.1%)	Spain	28.40 ± 3.34/Overweight 25–29.9 (59.2%) >30 (24.5%)	[42]
663	64 (56–72)	F (33.1%)/M (66.8%)	Spain	28.3 (25.5–32.2)/Overweight	[43]
15,111	69.4 (18–102)	F (42.8%)/M (57.2%)	Spain	>30 (21.2%)	[44]
118	60.16	F (44.1%)/M (55.9%)	Spain	29.7 ± 5.79/Overweight >30.0 (41%) >25.0–29.9 (37.3%)	[45]
242	64 (56–71)	F (18.2%)/M (80.2%)	Italy	27.7 (25.4–29.7)/Overweight	[46]
50	50.5	F (30%)/M (70%)	Mexico	28 ± 3/Overweight	[47]

^a^ Common type; ^b^ severe cases; ^c^ group of patients with symptom onset for 10 days or less; ^d^ group of patients with symptoms more than 10 days; ^e^ general group; ^f^ critical group; ^g^ non-severe patients; ^h^ severe patients; ^I^ patients with intubation; ^j^ patients without intubation; ^k^ stable, non-severe; ^l^ progressively severe; patients admitted in intensive care who required invasive mechanical ventilation ^m^ and those who did not ^n^.

**Table 4:** Summary of articles with data indicating obesity, but not BMI, and patients affected by COVID-19.

No of Patients	Age	Sex	Country	Anthropometric Parameters	References
1	73	F	USA	Obesity	[49]
33	41.8 ± 14.1	F (48.5%)/M (51.5%)	China	9.1% obesity	[50]
46	10–24	F (47.3%)/M (52.7%)	China	8.7% underweight 52.0% normal 37.0% overweight/obesity	[51]
129	54–100 ^a^ 22–79 ^b^ 52–88 ^c^	F (65.4%)/M (34.6%) ^a^ F (79.4%)/M (20.6%) ^b^ F (28.6%)/M (71.4%) ^c^	USA	33.3% obesity ^a^ 0% obesity ^b^ 3% obesity ^c^	[52]
1	59	F	Ecuador	Obesity class III	[53]

^a^ Resident, ^b^ healthcare personnel, and ^c^ visitor in a long-term residential care facility in Washington.
